# Successful chelation in beta-thalassemia major in the 21^st^ century

**DOI:** 10.1097/MD.0000000000035455

**Published:** 2023-10-13

**Authors:** Christina Fragodimitri, Vasiliki Schiza, Anastasios Giakoumis, Kalliopi Drakaki, Anastasia Salichou, Fotis Karampatsos, Jacqueline Yousef, Markissia Karageorga, Vasili Berdoukas, Athanasios Aessopos

**Affiliations:** a Thalassemia Unit, “Aghia Sofia” Children’s Hospital, Athens, Greece; b Thalassemia Unit, “G. Gennimatas” General Hospital, Athens, Greece; c Thalassemia Unit, “Lefkos Stavros” Hospital, Athens, Greece; d 1^st^ Academic Department of Internal Medicine, “Laiko” General Hospital, Athens, Greece.

**Keywords:** iron chelation therapy, iron overload, magnetic resonance imaging, thalassaemia

## Abstract

This century has seen a revolution the management of beta-thalassemia major. Over a 12-year period to 2016, we aimed to analyze the benefits of such advances. In 209 patients, independent of the chelation regimen, ferritin, cardiac T2* and liver iron concentration changes were evaluated. We defined chelation success (ChS) as no iron load in the heart and acceptable levels in the liver. Over 3 early magnetic resonance imagings, the same parameters were assessed in 2 subgroups, the only 2 that had sufficient patients continuing on 1 regimen and for a significant period of time, 1 on deferrioxamine (low iron load patients n = 41, Group A) and 1 on deferoxamine-deferiprone (iron overloaded n = 60, Group B). Finally, 28 deaths and causes were compared to those of an earlier period. The 209 patients significantly optimized those indices, while the number of patients with chelation success, increased from 6% to 51% (*P* < .0001). In group A, ChS after about 8 years increased from 21 to 46% (*P* = .006), while in Group B, from 0% to 60% (*P* < .001) after about 7 years. Deaths over the 2 periods showed significant reduction. Combined clearance of cardiac and liver iron (ChS) is feasible and should become the new target for all patients. This requires, serial magnetic resonance imagings and often prolonged intensified chelation for patients.

## 1. Introduction

The implementation of magnetic resonance imaging (MRI) to determine the amount of iron in vital organs, mainly heart and liver,^[1]^ and the availability of oral iron chelators, deferiprone (DFP), deferasirox (DFX)^[2]^ in addition to parenteral deferoxamine (DFO), have revolutionized the management of Thalassaemia Major patients (TM). The 3 iron chelators, as well as their combinations, offered a variety of choices affecting health care providers’ decisions on prescribing chelation according to MRI results.^[3]^

In Greece, up until the start of this century, with TM, we lived a long period with early death of these patients, with heart failure as the main cause, causing deep pain for relatives and health care professionals. With the above new applications in our country, for the first time we saw a rapid conversion of congestive heart failure and restoration of cardiac function in patients after administration of the DFO- DFP combination.^[4]^ Also, in a study of that combination, alongside heart-liver iron reduction, improvement of thyroid, gonadal, and pancreas function with reduction of pharmaceutical interventions for hypothyroidism, hypogonadism and diabetes mellitus were reported.^[5]^

Thus, in our unit, having a clear knowledge of the risks of iron load of these organs and the possible improvement with medication, we set the main goal in each patient to clear their hearts and livers of iron, based on their MRI results using the 3 chelators. The administration of the above *c*ombination, the only 1 that was being implemented at that time, was principally prescribed in patients, with a view to achieve relatively rapid iron clearance.

In this retrospective study, from data collected between 2004 and 2016, we analyzed the long-term benefits, in routine practice, of these advances on TM, regarding heart and liver iron load. We also planned to evaluate the efficacy of each chelator regimen and record the deaths and the main causes thereof. Our hypothesis was that, with the results of this evaluation, we would be able to have some useful informative conclusions for patients treatment modifications, to maximize the improvement in their iron status and reduce morbidities.

## 2. Patients and methods

A total of 330 patients with TM were followed at our center. The median age of these patients was 44 years (range: 7–62.5 years). Transfusions were approximately every 14 days according to standard recommendations.^[6,7]^ For their chelation therapy the dose of DFO was between 40 to 50 mg/kg/day by subcutaneous infusion for 5 to 7 days per week for monotherapy, DFP was at 75 mg/kg/day in 3 divided doses. The dose of dispersible DFX was between 10 to 40 mg/kg/day. In some cases, the recommended chelation regimen had to be changed because of adverse effects from the chelator(s) prescribed. These recommendations were in accordance with standards and guidelines and published data.^[2,6,7]^ At each transfusion, the center’s physicians recorded their recent history and pre-transfusion Hb levels including medication history. Annual adherence was expressed as a percentage of actual use/recommended use.^[8]^ Ferritin levels were measured every 2 to 3 months, cardiological evaluation with ECG, Echo-Doppler and clinical examination were undertaken in all patients at least annually and more often if deemed necessary.

The study started when MRI iron load evaluation became available in our country after 2003. The same physicist interpreted all the MRI studies. Patients were referred for MRI according to perceived concern and as some reached adolescence. The interval between MRIs was determined by the patients clinical status. Chelation changes were made according to their results, targeting organ iron clearance to acceptable levels. Acceptable iron levels were regarded as cardiac T2* being > 22msec and liver iron <3 mg/g dry weight. We refer to this as chelation success (ChS) (explained further below). Patients with mild to moderate iron load on MRI, might have had 1 of the 3 chelators prescribed as monotherapy, based on their different modes of action, potential side effects and possible improvement in their acceptance of therapy. The combination was prescribed in patients with severe iron load of the heart, or liver or both. Those patients with moderate iron load, who were on monotherapy in between MRIs, when their subsequent MRI did not show improvement, were also recommended to be on combination of DF0-DFP. For patients on that combination, DFP was prescribed daily. DFO tended initially to be 5 days per week and was reduced to 2 to 3 times per week or stopped completely once ChS was achieved and monotherapy with one of the 3 chelators could be continued. The distribution of the patients’ chelation during the time intervals between their first 3 MRIs is shown in Flow Table 1.

**Table 1:** This flow table shows the number of patients on a particular chelation regimen for their first 3 MRIs and the mean time interval between them, starting at their first. As the patients had subsequent MRIs, they were changed to different regimens according to whichever regimen was considered optimal for their management. It is shown as a flow diagram, rather than just numbers in each regimen.

Time (yr)	MRI1 (0)	MRI2 (3.6386)	MRI3 (7.0171)
Number of patients on different chelation regimes	247 DFO	107 DFO	41 DFO (group A)
			41 DFO + DFP
			9 DFX
		97 DFO + DFP	60 DFO + DFP (group B)
			8 DFO
			2 DFX
			1 DFP
		14 DFX	5 DFO + DFP
			3 DFX
			1 DFO
			1 DFP
		10 DFP	4 DFO + DFP
			3 DFP
			2 DFX
			1 DFO
	13 DFP	5 DFO + DFP	2 DFO + DFP
			1 DFO + DFX
			1 DFX
		3 DFO	3 DFX
		3 DFP	2 DFO + DFP
			1 DFP
		1 DFX	-
	3 DFO + DFP	2 DFO + DFP	2 DFO + DFP

DFO = desferrioxamine, DFO + DFP = combination therapy with desferrioxamine and deferiprone, DFP = deferiprone, DFX = deferasirox, MRI = magnetic resonance imaging.

The overall analysis was independent of the chelation regimen prescribed. The criteria for inclusion were that their first and last MRI findings were at least 5 years apart. The parameters evaluated were mean ferritin for the year before the first MRI and final MRI, MRI-acquired left ventricular ejection fraction, cardiac T2*, and liver iron concentration (LIC).

For the subgroup analysis, the criteria were that there be more than twenty patients on a particular regimen and who had had 3 consecutive MRIs while remaining on a consistent chelation regimen. We aimed to determine the grouped effect of the different chelation regimens according to each individual patient’s chelation therapy. The parameters evaluated were the same as for the overall analysis.

In the 330 original patients we retrospectively recorded the deaths over 2 similar periods in our unit, 1992 to 2004 and 2004 to 2016 and compared the total number and causes of deaths in each period.

A cardiac-dedicated General Electric Magnetometer (1.5 T magnet – Signa CVI with 40 mT/m gradients and appropriate cardiac software; General Electric, Milwaukee, IL) was used for the MRI measurements. The calculation of the absolute concentration of iron in the liver was based on the Wood equation: [Fe] = 0.202 + 0.0254 multiplied by the R2*(in hertz) where R2* = 1000/T2*.^[9]^ Cardiac statistics were performed using the mean of the mapped cardiac T2* values. Left ventricular ejection fraction and volumes, as determined by the MRI, were also recorded.

In our unit Cardiac T2* < 8 msec was categorized as heavy iron load, 8 to 14 msec as moderate, 14 to 22 msec as mild and > 22msec as iron-free. For liver iron concentration, < 1.5 mg/g dry weight (dw) was regarded as iron-free, 1.5 to 7.0 as mild, 7.0 to 14.0 as moderate and over 14.0 as severe, slightly modified from the criteria of Wood and Ghugre.^[10]^ Additionally, even though < 1.5 is normal, the UK standards^[5]^ suggested that an optimal range for LIC should be between 3 to 7 mg/g dry weight. Our unit’s policy was that a LIC < 3 was acceptable and desirable, reflecting a successful liver clearance. If patients reached such levels and their cardiac iron was also > 22msec, they were regarded as having achieved the target of ChS.

### 2.1. Statistical analyses

Statistical analyses were performed using MedCalc v19 and GraphPad Prism v8. As all analyzed parameters did not display a normal distribution, results are presented as median values with their corresponding 2-tailed probabilities (95% confidence intervals are shown in respective figures), and comparisons involved nonparametric rank sum tests for paired samples (Wilcoxon test) and independent samples (Mann-Whitney test). For the comparison of categorical data between time periods and/or patient groups, Chi-squared, McNemar, and Cochran Q test were utilized. Statistical significance was set at a *P* value < .05. In the overall analysis MRI parameters were compared using the Wilcoxon paired samples test, and for the comparison of ChS, McNemar test was utilized. Regarding the subgroup analysis, groups were compared at each MRI as independent samples with Mann-Whitney test, and each group was also paired with itself between subsequent MRIs and compared with the Wilcoxon test for progression within each therapy group. When comparing ChS for each patient group between MRIs, Cochran Q test was performed, while ChS comparisons between patient groups at any MRI time point were based on the Chi-squared test. Death frequencies from any cause were compared between time periods with the Chi-squared test. Comparison of ages at death were carried out with Mann-Whitney test.

The study was approved for the anonymous publication of the data by the hospital’s Ethics Committee.

No funding was received from the unit in support of this accumulation of data nor for the preparation of the article.

Raw data are submitted as follows:

Data S1, Supplemental Digital Content, http://links.lww.com/MD/K125 of Deaths.

Data S2, Supplemental Digital Content, http://links.lww.com/MD/K126 of patients in the overall analysis with names removed.

Data S3, Supplemental Digital Content, http://links.lww.com/MD/K127 of patients in the subset analysis with names removed.

## 3. Results

### 3.1. Overall analysis

**Figure 1.**
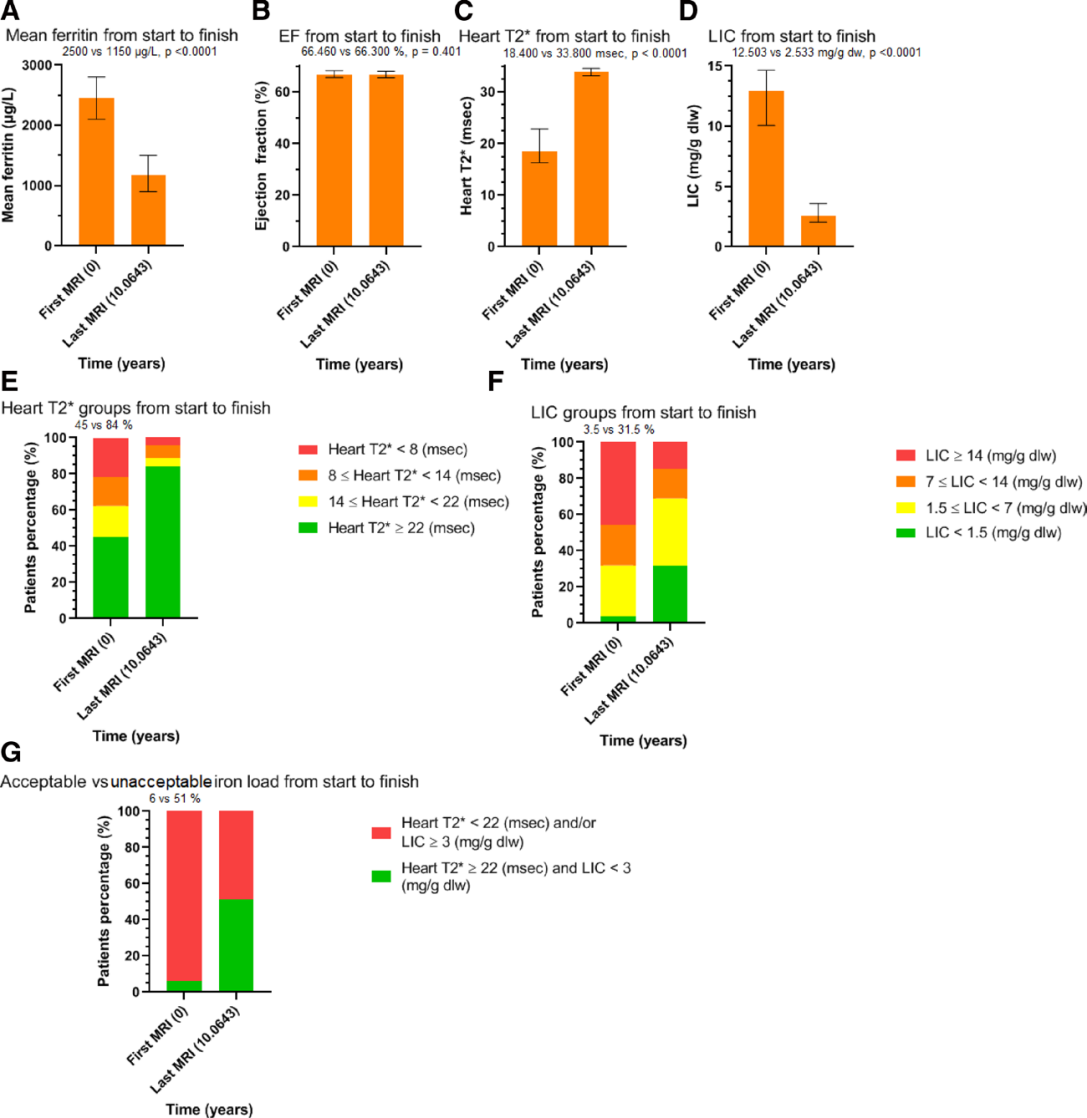
(A–G) Showing graphically, the results of the studied parameters changes, between first and last MRI in the overall analysis. The median time interval in years is shown in the last column. MRI = magnetic resonance imaging.

From the total 330 patients, 28 died during the study period and did not fulfill the criteria for the main analysis and were evaluated in the death analysis of the study. Also, 58 patients did not fulfill the criteria of having either enough MRIs or the proscribed time between them, while 35 patients moved to other centers. Thus, 209 patients, fulfilled the criteria for this analysis. They had a median age at the time of their first MRI of 28 years (range: 18 to 46.5 years). Table 2, Table S1, Supplemental Digital Content, http://links.lww.com/MD/K128, shows the analysis of the 209 patients. Figure 1A and D, derived from that table, show graphically, their results and the clear improvement of the 3 indices assessed. Figure 1E and F, depict separately, the percentage of patients with iron-free heart and acceptable liver changes over the study period (45 vs 84% and 3.5 vs 31.5%) respectively, and the mean time in years of the interval between first and last MRI. Moderate and severe iron loaded patients reduced their load substantially in both organs.

**Table 2:** Comparison of paired samples (Wilcoxon test) for parameters and McNemar test comparison of ChS between first and last MRI

Paired variables (n = 209)	First MRI	Last MRI	Difference	*P* value
Median mean ferritin (μg/L)	2500	1150	−779	<.0001
Median ejection fraction (%)	66.460	66.300	−0.505	.401
Median heart T2* (msec)	18.400	33.800	10.600	<.0001
Median LIC (mg/g dw)	12.503	2.533	−8.114	<.0001
ChS (%)	6	51	45	<.0001

ChS = chelation success, LIC = liver iron concentration, MRI = magnetic resonance imaging, dw = dry weight.

Figure 1 G shows the number of patients with ChS, which increased from 6% to 54%.

In general, therapy was well tolerated. Severe side effects were noted in only 10 patients: 3 patients had allergic reactions to DFO and one of these was on combination therapy with desferrioxamine and deferiprone (DFO + DFP), leucopenia occurred in 6 patients on DFP of whom 4 were on DFO + DFP and 1 patient developed significant proteinuria on DFX. No patients on DFP developed agranulocytosis. No patients died because of side effects. Acceptance of therapy increased from between 50 to 65% before, to over 80% subsequently.

### 3.2. Subgroup analysis

Of the 209, who had an initial MRI, 196 were on DFO monotherapy, 10 were on DFP and 3 on DFO + DFP (column 1, Flow Table 1). The distribution of the chelation regimens thereafter and the number of patients that received them are shown over the subsequent columns of Flow Table 1 corresponding to their MRIs and the mean time interval between scans.

**Figure 2.**
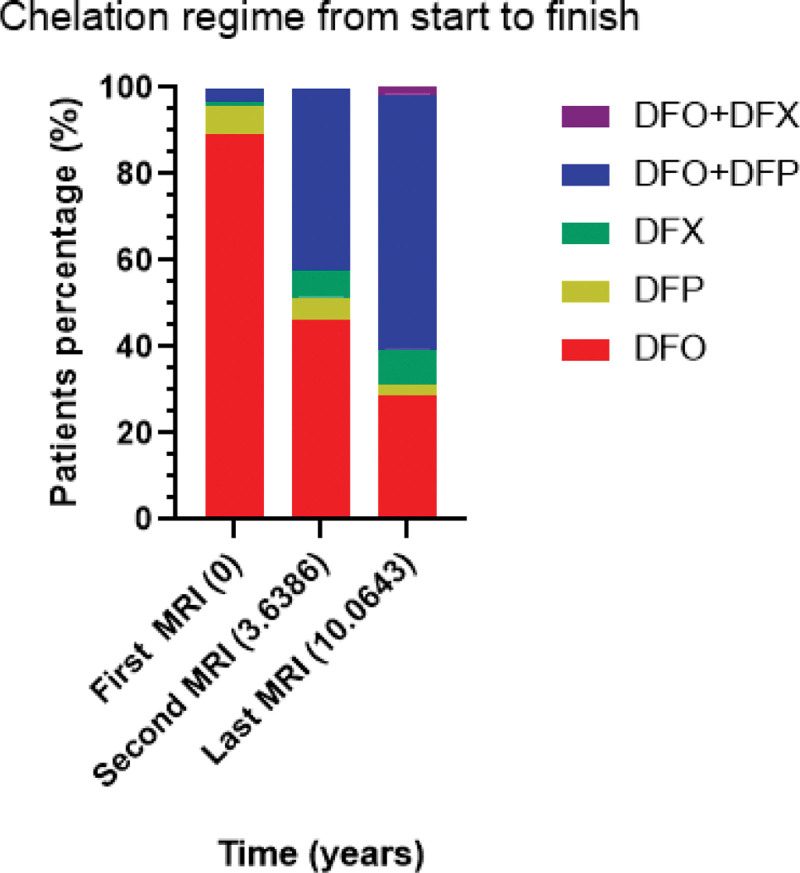
Shows the percentage of the 209 patients on different chelation regimens at the median time of each of the 3 MRIs performed. MRI = magnetic resonance imaging.

The prescribed therapy over the first 3 MRIs for the 209 patients is shown in Figure 2 and column 2 and 3 of Flow Table 1 shows which patients continued the different regimens over that time. Gradually, DFO + DFP was the most common regimen prescribed. At the second MRI, 96 patients were on DFO (46%), 11 on DFP (5.5%), 13 on DFX (6%) and 89 were on DFO + DFP (42.5). Finally, at the 3rd MRI the numbers were 61 (29%), 5 (2.5%), 17 (8%) and 123 (59%) respectively. The remainder 1.5% (3 patients) were on the DFO + DFX combination.

Flow Table 1 also demonstrates that there were insufficient patients on monotherapy with DFP or DFX, who remained for an adequate period on a consistent regimen for a subgroup analysis.

Based on the above distribution of chelation regimens, the patients, who were consistently on DFO from MRI 1 to 3 were Group A (n = 41) and those who continued DFO + DFP after switching from DFO, were Group B (n = 60). Group A patients had a median age of 28 years (range: 12.5–44.5 years) at the time of their first MRI, while Group B, 30 years (range: 9.5–41.5 years).

**Figure 3.**
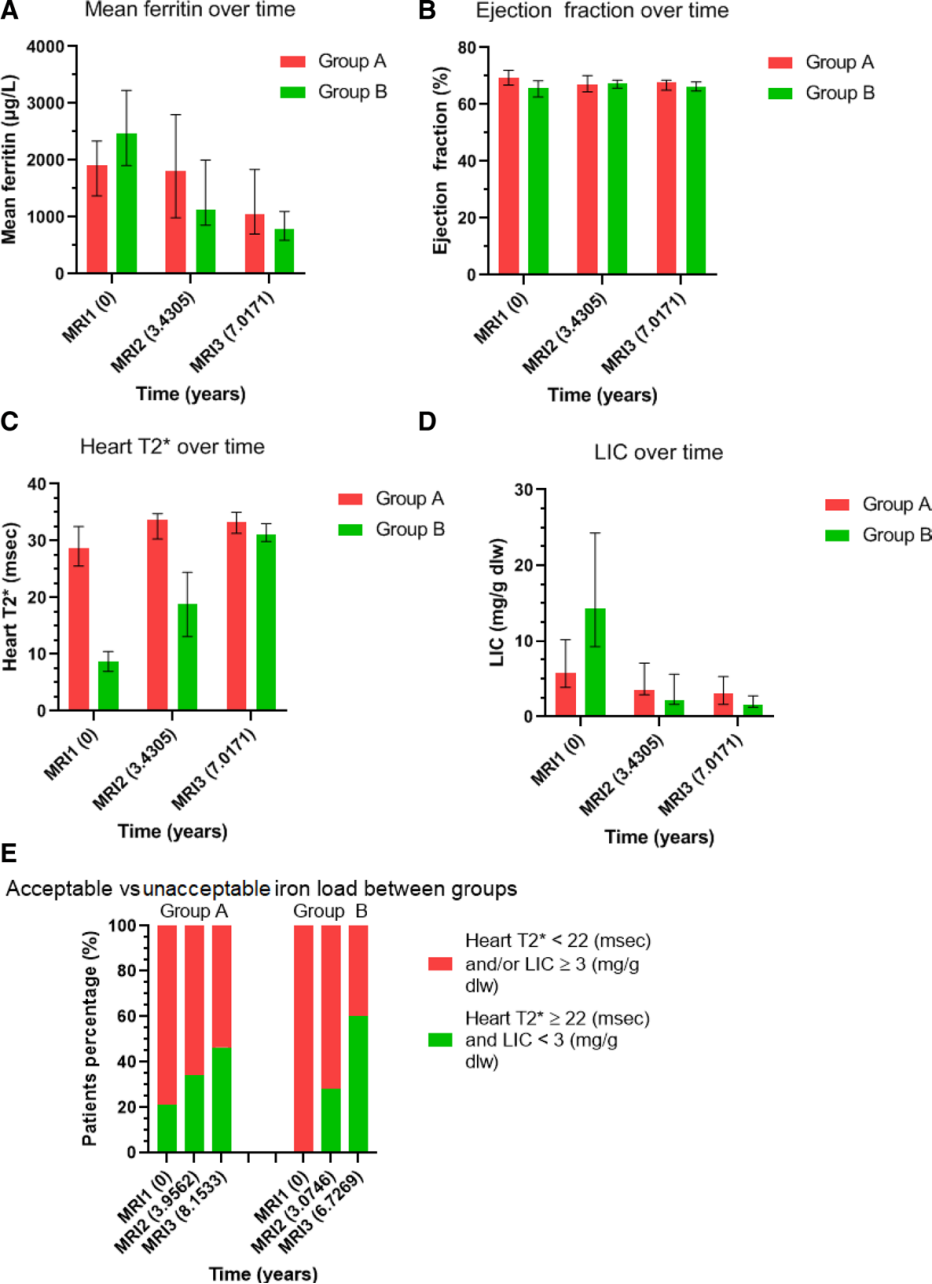
(A–E) Showing graphically, the comparison of independent samples between Group A and Group B at consequent MRI studies. Median time interval in years for both groups between scans is shown is shown in figures (A–D) and the median time interval for each group is shown in (E). MRI = magnetic resonance imaging.

Table 3, Table S2, Supplemental Digital Content, http://links.lww.com/MD/K129, analyses parameters for Group A and Table 4, Table S3, Supplemental Digital Content, http://links.lww.com/MD/K130, those of Group B. Table 5, Table S4, Supplemental Digital Content, http://links.lww.com/MD/K131, compares parameters between Group A and Group B. Figure 3A and E are derived from these tables.

**Table 3:** Group A. Comparison of paired samples (Wilcoxon test) for parameters and Cochran Q test comparison of ChS between consecutive MRIs.

Paired variables for Group A (n = 41)	MRI1	MRI2	Difference	*P* value
Median mean ferritin (μg/L)	1919	1800	−67	.669
Median ejection fraction (%)	69.3	67.150	−2.110	.059
Median heart T2* (msec)	28.600	33.600	2.200	.039
Median LIC (mg/g dw)	5.744	3.544	−1.429	.058
ChS (%)	21	34	13	>.05
	MRI2	MRI3	Difference	*P* value
Median mean ferritin (μg/L)	1800	1058	−216.250	.074
Median ejection fraction (%)	67.150	67.660	0.500	.643
Median heart T2* (msec)	33.600	33.300	0.400	.468
Median LIC (mg/g dw)	3.544	3.122	−1.333	.014
ChS (%)	34	46	12	>.05
	MRI1	MRI3	Difference	*P* value
Median mean ferritin (μg/L)	1919	1058	−368	.084
Median ejection fraction (%)	69.3	67.660	−3.035	.712
Median heart T2* (msec)	28.600	33.300	2.950	.012
Median LIC (mg/g dw)	5.744	3.122	−2.297	.003
ChS (%)	21	46	25	.006

ChS = chelation success, LIC = liver iron concentration, MRI = magnetic resonance imaging, dw = dry weight.

**Table 4:** Group B. Comparison of paired samples (Wilcoxon test) for parameters and Cochran Q test comparison of ChS between consecutive MRIs.

Paired variables for Group B (n = 60)	MRI1	MRI2	Difference	*P* value
Median mean ferritin (μg/L)	2460	1125	−882	<.0001
Median ejection fraction (%)	65.630	67.475	2.428	.082
Median heart T2* (msec)	8.700	18.800	9.090	<.0001
Median LIC (mg/g dw)	14.313	2.127	−9.500	<.0001
ChS (%)	0	28	28	<.001
	MRI2	MRI3	Difference	*P* value
Median mean ferritin (μg/L)	1125	780	−308.250	.003
Median ejection fraction (%)	67.475	66.300	−1.090	.100
Median heart T2* (msec)	18.800	31.100	8.950	<.0001
Median LIC (mg/g dw)	2.127	1.578	−1.272	.0001
ChS (%)	28	60	32	<.001
	MRI1	MRI3	Difference	*P* value
Median mean ferritin (μg/L)	2460	780	−1269.250	<.0001
Median ejection fraction (%)	65.630	66.300	0.795	.583
Median heart T2* (msec)	8.700	31.100	18.950	<.0001
Median LIC (mg/g dw)	14.313	1.578	−13.586	<.0001
ChS (%)	0	60	60	<.001

ChS = chelation success, LIC = liver iron concentration, MRI = magnetic resonance imaging, dw = dry weight.

**Table 5:** Group A and Group B. Comparison between independent samples (Mann–Whitney test) for parameters and Chi-squared test comparison of ChS at MRI1, MRI2 and MRI3.

Variables	Group A (n = 41)	Group B (n = 60)	Difference	*P* value
Median interval MRI1–MRI2 (yr)	3.956	3.075	−0.705	.024
Median interval MRI1–MRI3 (yr)	8.153	6.727	−0.461	.356
Median mean ferritin at MRI1 (μg/L)	1919	2460	461.500	.047
Median mean ferritin at MRI2 (μg/L)	1800	1125	−300	.190
Median mean ferritin at MRI3 (μg/L)	1058	780	−250	.116
Median ejection fraction at MRI1 (%)	69.300	65.630	−4.520	.007
Median ejection fraction at MRI2 (%)	67.150	67.475	0	1
Median ejection fraction at MRI3 (%)	67.660	66.300	−0.660	.541
Median heart T2* at MRI1 (msec)	28.600	8.700	18.600	<.0001
Median heart T2* at MRI2 (msec)	33.600	18.800	−10.800	<.0001
Median heart T2* at MRI3 (msec)	33.300	31.100	−1.700	.108
Median LIC at MRI1 (mg/g dw)	5.744	14.313	6.857	.001
Median LIC at MRI2 (mg/g dw)	3.544	2.127	−0.846	.130
Median LIC at MRI3 (mg/g dw)	3.122	1.578	−0.378	.089
ChS at MR1 (%)	21	0	−21	<.0001
ChS at MR2 (%)	34	28	−6	.227
ChS at MR3 (%)	46	60	14	.0278

ChS = chelation success, LIC = liver iron concentration, MRI = magnetic resonance imaging, dw = dry weight.

For Group B, except for the median ejection fraction, which remained unchanged at the normal level through the MRI’s, all the other indices improved significantly in all paired MRI comparisons, being markedly better by MRI3. Similarly, for ChS, the increased number was very significant, even by the second MRI.

All B Group patients iron load indices, at the beginning, were significantly worse than those in Group A. Group B started showing significant improvement at MR2, and variables were not distinguishable from those in Group A at MRI3. The paired comparison of ChS frequencies between Group A and B with the Chi-squared test, showed that Group B started with negative significant differences (*P* < .0001) at MRI1, but concluded with superior outcomes to Group A, at MRI3 (*P* = .028). There was a significant difference in the time interval between scans in that Group B was shorter than Group A.

Figure 3A and E, shows Group A patients, starting with less iron load, slowly improved their analyzed indices through to the 3 MRI’s.

Group B, the heavily overloaded patients, even by MRI2, showed dramatic improvement in their iron load indices. While by the third MRI3 their results were statistically significantly better than those of Group A patients (Fig. 3E and Table 5 Table S4, Supplemental Digital Content, http://links.lww.com/MD/K131).

### 3.3. Analysis of deaths

The number of patients attending the unit between 1992 to 2004 was similar those attending between 2004 to 2016 (338 vs 330). The median age of all 54 patients who died in the former group was 24 years (range:8–38 years) and of 28 patients who died during the latter period, it was 39.5 years (range: 19–52 years). In those 28, 16 patients died of congestive heart failure (CHF), 7 of hepatocellular cancer (HCC) and 5 from other causes. All deaths from cardiac causes, during the second period, occurred only in the first haft of that period, between 1 to 4 years from the first MRI., (mean 2,7msec) and did not have the opportunity to have enough MRIs to fulfill the required criteria.

**Figure 4.**
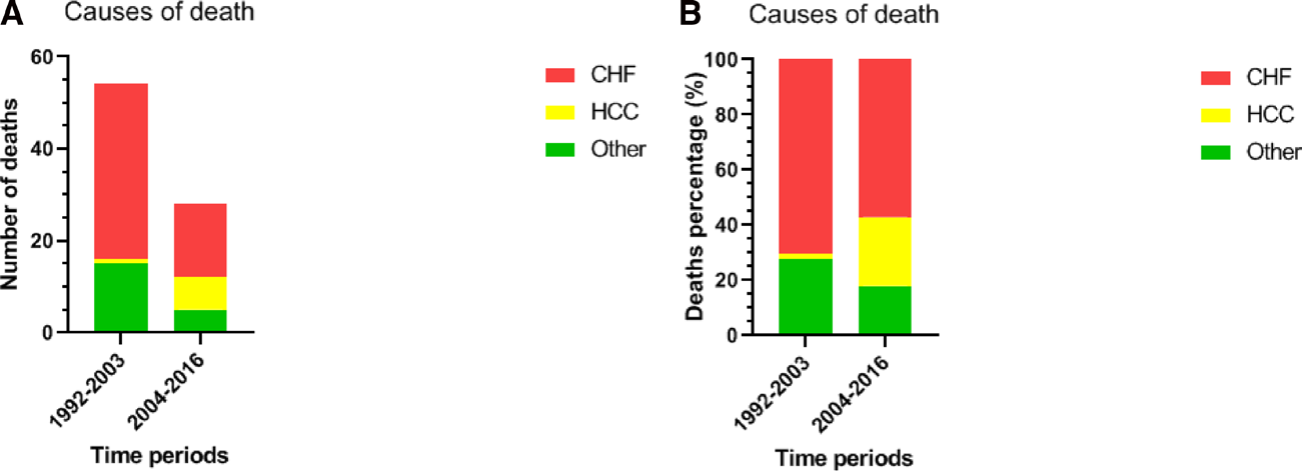
(A–B) showing graphically, the Comparison of two periods regarding causes of death in absolute number and percentage.

Table 6, Table S5, Supplemental Digital Content, http://links.lww.com/MD/K132 shows the Chi-squared test comparison for death frequencies, causes and comparison between independent samples (Mann–Whitney test) for ages at death between time periods. This is the basis for Figure 4A and B. The total deaths and those due to CHF as well as to other causes, decreased significantly between the 2 time periods, in contrast to deaths due to HCC, which increased significantly. The median age of death increased significantly (24 vs 39.5 years), and this was also noted separately, in all 3 causes of death (Fig. 4A and B).

**Table 6:** Chi-squared test comparison for death frequencies causes and comparison between independent samples (Mann–Whitney test) for ages at death between time periods.

All deaths	1992–2004	2004–2016	Difference [relative proportion]	*P* value
Patients	54	28	26 [≈2:1]	.004
Median age (yr)	24	39.5	14	<.0001
CHF deaths	1992–2004	2004–2016	Difference [relative proportion]	*P* value
Patients (percentage)	38 (70%)	16 (57%)	22 [≈+20%]	.003
Median age (yr)	24.5	31.5	7	.004
HCC deaths	1992–2004	2004–2016	Difference [relative proportion]	*P* value
Patients (percentage)	1 (2%)	7 (25%)	−6 [≈−1200%]	.034
Median age (yr)	33	44	-	-
Other deaths	1992–2004	2004–2016	Difference [relative proportion]	*P* value
Patients (percentage)	15 (28%)	5 (18%)	10 [≈+33%]	.025
Median age (yr)	23	41	20	.001

CHF = congestive heart failure, HCC = hepatocellular carcinoma.

## 4. Discussion

The most important and valuable observations in our study, was the excellent outcome that resulted from the new modalities of monitoring, more options in chelation and the therapy chosen. With these advances, we were able to achieve chelation success in a large percentage of our patients in both the overall and subgroup analysis. In parallel, there was a significant reduction in deaths. The markedly improved acceptance of therapy also contributed to these excellent outcomes.

Though many patients at baseline had a detectable cardiac iron burden (heartT2*: 18.4 msec) and increased liver iron (LIC: 12.50 mg/g dw), most were able to successfully de-iron both organs over the observation period (Fig. 1E and F). Patients with iron-free hearts, almost doubled proportionately (from 45% to 84%). One hundred sixty-seven of 209 studied patients achieved clearance of heart iron. Since cardiac dysfunction due to iron load in TM, remains the main cause of heart failure and death, such outcomes render our patients relatively risk-free from cardiac morbidity and mortality. Heart function was maintained at normal levels in all patients over a mean period of 10 years, indicating good prospects for heart function in the future. In the past, it would have been expected that some patients will have deterioration of cardiac function over such a period, considering their increasing age.^[11]^

The median ferritin level was significantly reduced from 2500μg/L to 1150 μg/L. Patients with acceptable liver iron, increased almost tenfold (3.5% vs 31.5%). The number of patients with moderate and heavy iron load decreased, also leaving a large percentage of patients in the mildly iron loaded group (Fig. 1E and F). Liver iron load contributes to cirrhosis and cancer development in TM,^[12,13]^ thus, reduction of liver iron could also reduce the risk of liver morbidities.

Impressively, patients showed an almost 9-fold increase (6% vs 54%) in ChS in that 106 of 209 patients in this study achieved this outcome (Fig. 1G). Such results and even better, seem to be feasible by appropriate treatment modification if these goals were recognized as the target for TM management.

A few other studies have demonstrated similar outcomes with respect to cardiac clearance, in routine clinical practice, but not for such long periods and with fewer patients. Very few have shown such low levels of liver iron.^[5,14,15]^

In the subgroup analysis, Group A patients continued their DFO treatment, due to their good initial MRI results. Figure 3A and D shows the slow optimization of their indices. Those patients showed 21% ChS at the first MRI, while at the third MRI, it increased to 46.5% (Fig. 3E), indicating mainly the slow liver iron reduction for those patients, since the heart iron load was within normal limits from the start and was maintained clear of iron. The slow liver iron removal is expected in patients who start with relatively low liver iron. Thus, considering this action of DFO and the other 2 chelators, if early MRIs were to show no or low levels of iron load, monotherapy, with any of the 3 chelators, could be enough to prevent iron load and organ injury.

Group B who at first MRI showed moderate cardiac overload, and some even with suboptimal ejection fraction, and severe liver iron overload, with increased median ferritin levels, showed better progress compared to Group A. Through a period of approximately 3 years (second MRI) and 7 years (third MRI), they managed to get statistically significant improvement in all the above indices, in each one of the 2 periods (Fig. 3), even though the time periods were shorter than those for Group A (Fig. 3E). Of note the greatest improvement occurred in the first period. Both heart and liver, were overloaded, particular the latter (MRI1 Τ2*:8.7-LIC:14.313 mg/g dw) and were close to normal (Τ2*:18.800- LIC:2.098 mg/g dw) by MRI2. The former removed them from a danger zone, with respect to cardiac iron. In some of the Group B patients, who showed clear iron reduction by MRI2 (Fig. 3Ε), the frequency of DFO might have been reduced to 2 to 3 days/week. A few patients, after the first MRI, showed ChS at the second MRI, and their treatment was replaced with monotherapy. As these patients were not on DFO + DFP between the 2^nd^ and 3^rd^ MRI, they were included only in the overall analysis.

At the first MRI no Group B patients had achieved ChS, by the second, 28 % had and by the third MRI, 60% had (Fig. 3Ε). In addition, as discussed above, patient’s heart and liver iron load were close to normal already by MRI2. Thus, a period of 3 years of DFO + DFP seems to be a critical time to unload severe iron load patients and the 28% of those who showed ChS, could have been treated less intensively after MRI2.

The significant reduction of the total deaths in comparison to previous decade was a very important finding. Furthermore, the median age of death increased significantly (24 vs 39.5 years), and this was also noted separately, in all 3 causes of death (Fig. 4A and B). As deaths occurred only in the first half of the 2004 to 2016 period, it, reflects that aiming for ChS and the ability to achieve it, is an appropriate goal.

With respect to premature cardiac deaths, of the 16 patients who died of CHF, all but 1 had severe iron load (median cardiac T2* of 3.9 msec). The main reason for not being able to save them was because of difficulties, particularly psychological, with accepting the chelation therapy regimens. Despite our experience that intensive chelation may reverse even the most severe cardiac iron status,^[16]^ we were unsuccessful in persuading the 15 who died, to accept intensification. The one, with clear heart iron load, despite her excellent compliance, had residual heart damage after severe iron load and prolonged heart failure 20 years before and never completely recovered.

As the patients age, liver disease becomes a major issue.^[17]^ The percentage of deaths, due to liver malignancies increased proportionately by almost thirteenfold (1 case vs 7, 2% vs 25%) (Fig. 4A and B). The median age of death for these 7 patients was 44 years while that in the earlier group the 1 patient who died was 33 years old.

Viral hepatitis plays a significant role and all patients in this study who died of hepatic cancer had past infection with hepatitis C. Additionally, liver iron overload and persistent circulating free iron increase the risk of hepatic malignancy.^[18]^ Bringing patient’s LIC down to acceptable levels and consistent exposure to chelation may be beneficial in prevention of malignancy.^[19]^ Consistent exposure may also play a role in reducing the oxidant activity of free iron with improved benefit to many organs.^[19]^

Patients with HCC in this study, went through long periods during which transfused blood could not be adequately tested for hepatitis and only moderate DFO chelation therapy was prescribed. At the first MRI, the median LIC measurements were 8.669 mg/g dw. The excessive liver ion load and the hepatitis explained the increasing frequency of HCC as they aged.^[13]^ Currently, hepatitis-free blood supply, significant advances in hepatitis treatment, along with early appropriate chelation therapy and consistent exposure to chelation especially in the younger populations may be beneficial in preventing hepatic malignancy in the future.

Clearly combination chelation is the best way to clear iron load of concern. Routine doses of the 2 chelators used for DFO + DFP, were well tolerated in most of the patients. In cases with difficulties in accepting this therapy, there are several other combinations (DFO + DFX and DFP + DFX) being used, as seen in Flow Table 1, with promising results (34). We have used those successfully, as a rescue intervention in some patients with severe heart iron load who present with leucopenia due to DFP and/or allergic reactions to DFO. Our results suggest the necessity for further and more extensive studies to be carried out using all other combinations to determine more powerful or equivalent alternative options, in severely iron loaded patients. DFO, the second most frequently used in our patients’ routine treatment, remains due to its good response, acceptance and addresses some patients’ concern regarding the complications associated with the oral chelators.

The limitations of our study are, the retrospective observational nature of the study, a bias toward intensive chelation therapy at a time when only 1 combination seemed available, resulting in an inability to have enough patients in other chelation regimens to allow more comparisons. In addition, the lack of subsequent MRI studies in patients who had only 1 MRI, an inherent bias, with respect to which chelation regimens were prescribed and that the deceased could not be included in the overall analysis. The latter might have shown worse overall results. The estimated adherence may not be accurate as patients do not always disclose the truth.

## 5. Conclusion

In this prolonged study, focused on the cardiac and liver iron clearance in TM, providing appropriate chelation under the guidance of the MRI studies, as well as increased patient compliance, allowed our unit to achieve extremely satisfactory iron clearance.

Importantly, the suggested approaches led also to a significant reduction in the premature deaths, as well as increased age at the time of death. Furthermore, no death was reported in the second half of this study period. Thus, this study showed that the desirable ChS target, is clearly feasible, offers clinical benefits and should become the treatment goal in TM.

## Acknowledgments

The authors wish to acknowledge the valuable contribution of Dr. Stathis Gotsis who evaluated all the MRIs of this study and the contribution of their late colleague Antonia Hatziliami.

## Author contributions

**Conceptualization:** Christina Fragodimitri, Anastasios Giakoumis, Markissia Karageorga, Vasili Berdoukas, Athanasios Aessopos.

**Data curation:** Vasiliki Schiza, Kalliopi Drakaki, Anastasia Salichou, Fotis Karampatsos, Jacqueline Yousef, Markissia Karageorga, Vasili Berdoukas, Athanasios Aessopos.

**Formal analysis:** Anastasios Giakoumis.

**Methodology:** Christina Fragodimitri, Vasiliki Schiza, Anastasios Giakoumis, Kalliopi Drakaki, Anastasia Salichou, Fotis Karampatsos, Jacqueline Yousef, Markissia Karageorga, Vasili Berdoukas, Athanasios Aessopos.

**Supervision:** Vasiliki Schiza, Athanasios Aessopos.

**Validation:** Anastasios Giakoumis.

**Writing – original draft:** Anastasios Giakoumis, Vasili Berdoukas, Athanasios Aessopos.

**Writing – review & editing:** Christina Fragodimitri, Vasiliki Schiza, Anastasios Giakoumis, Kalliopi Drakaki, Anastasia Salichou, Fotis Karampatsos, Jacqueline Yousef, Markissia Karageorga, Vasili Berdoukas, Athanasios Aessopos.

## Supplementary Material
