# Supplementary material for: Successful chelation in beta-thalassemia major in the 21st century
Source: Medicine (Baltimore). 2023 Oct 13;102(41):e35455. doi: 10.1097/MD.0000000000035455 (PMC10578721; doi:10.1097/MD.0000000000035455)
Supplement: Supplementary file 4 [file medi-102-e35455-s004.docx]

# **Table 1. Comparison of paired samples (Wilcoxon test) for parameters and McNemar’s test comparison of ChS between first and last MRI**

| Paired variables (n = 209) | First MRI | Last MRI | Difference | p value |
| --- | --- | --- | --- | --- |
| Median mean ferritin (μg/L) | 2500 | 1150 | **-779** | **<0.0001** |
| Median ejection fraction (%) | 66.460 | 66.300 | -0.505 | 0.401 |
| Median heart T2* (msec) | 18.400 | 33.800 | **10.600** | **<0.0001** |
| Median LIC (mg/g dw) | 12.503 | 2.533 | **-8.114** | **<0.0001** |
| ChS (%) | 6 | 51 | **45** | **<0.0001** |
