# Supplementary material for: Successful chelation in beta-thalassemia major in the 21st century
Source: Medicine (Baltimore). 2023 Oct 13;102(41):e35455. doi: 10.1097/MD.0000000000035455 (PMC10578721; doi:10.1097/MD.0000000000035455)
Supplement: Supplementary file 5 [file medi-102-e35455-s005.docx]

**Table2. Group A. Comparison of paired samples (Wilcoxon test) for parameters and Cochran’s Q test comparison of ChS between consecutive MRIs.**

| Paired variables for Group A (n = 41) | MRI1 | MRI2 | Difference | p value |
| --- | --- | --- | --- | --- |
| Median mean ferritin (μg/L) | 1919 | 1800 | -67 | 0.669 |
| Median ejection fraction (%) | 69.3 | 67.150 | -2.110 | 0.059 |
| Median heart T2* (msec) | 28.600 | 33.600 | **2.200** | **0.039** |
| Median LIC (mg/g dw) | 5.744 | 3.544 | -1.429 | 0.058 |
| ChS (%) | 21 | 34 | 13 | >0.05 |
|  | MRI2 | MRI3 | Difference | p value |
| Median mean ferritin (μg/L) | 1800 | 1058 | -216.250 | 0.074 |
| Median ejection fraction (%) | 67.150 | 67.660 | 0.500 | 0.643 |
| Median heart T2* (msec) | 33.600 | 33.300 | 0.400 | 0.468 |
| Median LIC (mg/g dw) | 3.544 | 3.122 | **-1.333** | **0.014** |
| ChS (%) | 34 | 46 | 12 | >0.05 |
|  | MRI1 | MRI3 | Difference | p value |
| Median mean ferritin (μg/L) | 1919 | 1058 | -368 | 0.084 |
| Median ejection fraction (%) | 69.3 | 67.660 | -3.035 | 0.712 |
| Median heart T2* (msec) | 28.600 | 33.300 | **2.950** | **0.012** |
| Median LIC (mg/g dw) | 5.744 | 3.122 | **-2.297** | **0.003** |
| ChS (%) | 21 | 46 | **25** | **0.006** |
