# Supplementary material for: Successful chelation in beta-thalassemia major in the 21st century
Source: Medicine (Baltimore). 2023 Oct 13;102(41):e35455. doi: 10.1097/MD.0000000000035455 (PMC10578721; doi:10.1097/MD.0000000000035455)
Supplement: Supplementary file 6 [file medi-102-e35455-s006.docx]

**Table3.Group B. Comparison of paired samples (Wilcoxon test) for parameters and Cochran’s Q test comparison of ChS between consecutive MRIs.**

| Paired variables for Group B (n = 60) | MRI1 | MRI2 | Difference | p value |
| --- | --- | --- | --- | --- |
| Median mean ferritin (μg/L) | 2460 | 1125 | **-882** | **<0.0001** |
| Median ejection fraction (%) | 65.630 | 67.475 | 2.428 | 0.082 |
| Median heart T2* (msec) | 8.700 | 18.800 | **9.090** | **<0.0001** |
| Median LIC (mg/g dw) | 14.313 | 2.127 | **-9.500** | **<0.0001** |
| ChS (%) | 0 | 28 | **28** | **<0.001** |
|  | MRI2 | MRI3 | Difference | p value |
| Median mean ferritin (μg/L) | 1125 | 780 | **-308.250** | **0.003** |
| Median ejection fraction (%) | 67.475 | 66.300 | -1.090 | 0.100 |
| Median heart T2* (msec) | 18.800 | 31.100 | **8.950** | **<0.0001** |
| Median LIC (mg/g dw) | 2.127 | 1.578 | **-1.272** | **0.0001** |
| ChS (%) | 28 | 60 | **32** | **<0.001** |
|  | MRI1 | MRI3 | Difference | p value |
| Median mean ferritin (μg/L) | 2460 | 780 | **-1269.250** | **<0.0001** |
| Median ejection fraction (%) | 65.630 | 66.300 | 0.795 | 0.583 |
| Median heart T2* (msec) | 8.700 | 31.100 | **18.950** | **<0.0001** |
| Median LIC (mg/g dw) | 14.313 | 1.578 | **-13.586** | **<0.0001** |
| ChS (%) | 0 | 60 | **60** | **<0.001** |
