# Supplementary material for: Successful chelation in beta-thalassemia major in the 21st century
Source: Medicine (Baltimore). 2023 Oct 13;102(41):e35455. doi: 10.1097/MD.0000000000035455 (PMC10578721; doi:10.1097/MD.0000000000035455)
Supplement: Supplementary file 7 [file medi-102-e35455-s007.docx]

**Table 4.Group A and Group B.** **Comparison between independent samples (Mann-Whitney test) for parameters and Chi-squared test comparison of ChS at MRI1, MRI2 and MRI3.**

| Variables | Group A (n = 41) | Group B (n = 60) | Difference | p value |
| --- | --- | --- | --- | --- |
| Median interval MRI1-MRI2 (years) | 3.956 | 3.075 | **-0.705** | **0.024** |
| Median interval MRI1-MRI3 (years) | 8.153 | 6.727 | -0.461 | 0.356 |
| Median mean ferritin at MRI1 (μg/L) | 1919 | 2460 | **461.500** | **0.047** |
| Median mean ferritin at MRI2 (μg/L) | 1800 | 1125 | -300 | 0.190 |
| Median mean ferritin at MRI3 (μg/L) | 1058 | 780 | -250 | 0.116 |
| Median ejection fraction at MRI1 (%) | 69.300 | 65.630 | **-4.520** | **0.007** |
| Median ejection fraction at MRI2 (%) | 67.150 | 67.475 | 0 | 1 |
| Median ejection fraction at MRI3 (%) | 67.660 | 66.300 | -0.660 | 0.541 |
| Median heart T2* at MRI1 (msec) | 28.600 | 8.700 | **18.600** | **<0.0001** |
| Median heart T2* at MRI2 (msec) | 33.600 | 18.800 | **-10.800** | **<0.0001** |
| Median heart T2* at MRI3 (msec) | 33.300 | 31.100 | -1.700 | 0.108 |
| Median LIC at MRI1 (mg/g dw) | 5.744 | 14.313 | **6.857** | **0.001** |
| Median LIC at MRI2 (mg/g dw) | 3.544 | 2.127 | -0.846 | 0.130 |
| Median LIC at MRI3 (mg/g dw) | 3.122 | 1.578 | -0.378 | 0.089 |
| ChS at MR1 (%) | 21 | 0 | **-21** | **<0.0001** |
| ChS at MR2 (%) | 34 | 28 | -6 | 0.227 |
| ChS at MR3 (%) | 46 | 60 | **14** | **0.0278** |
