# Supplementary material for: Successful chelation in beta-thalassemia major in the 21st century
Source: Medicine (Baltimore). 2023 Oct 13;102(41):e35455. doi: 10.1097/MD.0000000000035455 (PMC10578721; doi:10.1097/MD.0000000000035455)
Supplement: Supplementary file 8 [file medi-102-e35455-s008.docx]

**Table 5. Chi-squared test comparison for death frequencies causes and comparison between independent samples (Mann-Whitney test) for ages at death between time periods.**

| All deaths | 1992-2004 | 2004-2016 | Difference [relative proportion] | p value |
| --- | --- | --- | --- | --- |
| Patients | 54 | 28 | **26 [≈2:1]** | **0.004** |
| Median age (years) | 24 | 39.5 | **14** | **<0.0001** |
| CHF deaths | 1992-2004 | 2004-2016 | Difference [relative proportion] | p value |
| Patients (percentage) | 38 (70%) | 16 (57%) | **22 [≈+20%]** | **0.003** |
| Median age (years) | 24.5 | 31.5 | **7** | **0.004** |
| HCC deaths | 1992-2004 | 2004-2016 | Difference [relative proportion] | p value |
| Patients (percentage) | 1 (2%) | 7 (25%) | **-6 [≈-1200%]** | **0.034** |
| Median age (years) | 33 | 44 | - | - |
| Other deaths | 1992-2004 | 2004-2016 | Difference [relative proportion] | p value |
| Patients (percentage) | 15 (28%) | 5 (18%) | **10 [≈+33%]** | **0.025** |
| Median age (years) | 23 | 41 | **20** | **0.001** |
